# Supplementary material for: CFTR modulator therapy drives microbiome restructuring through improved host physiology in cystic fibrosis: the IMMProveCF phase IV trial
Source: Nat Commun. 2025 Nov 18;16:10111. doi: 10.1038/s41467-025-64218-z (PMC12627475; doi:10.1038/s41467-025-64218-z)
Supplement: Supplementary file 1 — Supplementary Information [file 41467_2025_64218_MOESM1_ESM.pdf]

Supplementary Information

CFTR modulator therapy drives microbiome restructuring through improved host physiology in cystic fibrosis: the IMMProveCF phase IV trial

Rebecca Luise Knoll[1,2,3,4,5], Melanie Meihua Brauny[6,7,8], Evelyn Robert[1], Louisa Cloos[1], Lydia Waser[1], Katja Hilbert[1], Nina Ulmer[6,7,8], Barlo Hillen[9], Till Birkner[2,3,4], Theda Ulrike Patricia Bartolomaeus[2,3,4,10], Oliver Nitsche[1], Víctor Hugo Jarquín-Díaz[2,3,4], Susan Lynch[5], Stephan Gehring[1], Lisa Maier[6,7,8], Krystyna Poplawska[1**], Sofia Kirke Forslund-Startceva[2,3,4,10,11*]

Inventory of Supplementary Information

1. Supplementary Figures 1-8
2. Supplementary Table S1

1. Supplementary Figures:

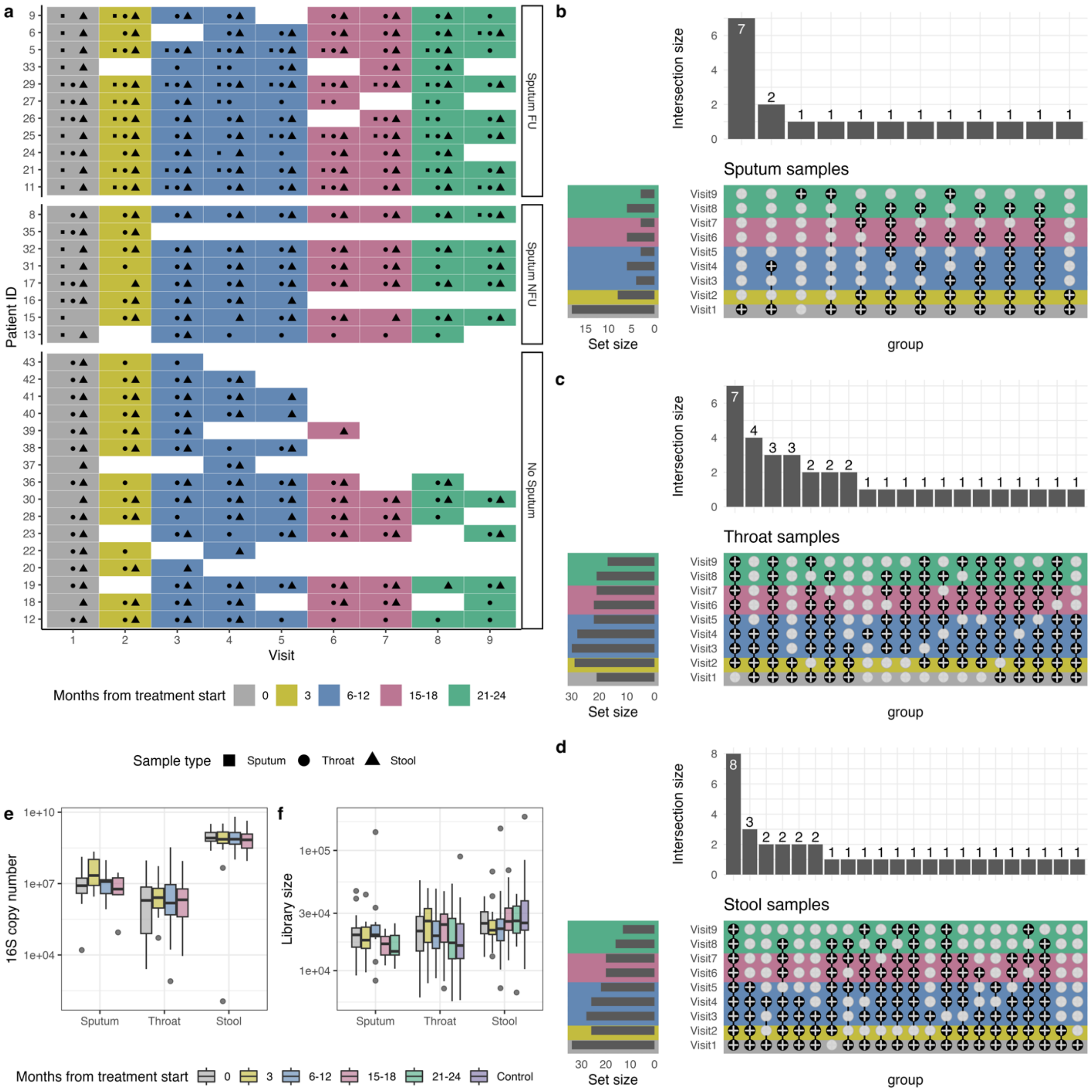

Supplementary Fig. 1

a, Overview of sample collection across study visits. Each shape denotes a sputum, throat, or stool sample at a given visit; background colors indicate months from ETI treatment start. Participants are grouped as: sputum with follow-up samples (Sputum FU), baseline sputum only (Sputum NFU), and no sputum (No Sputum).

b–d, Intersection plots for sputum (b), throat (c), and stool (d) showing numbers of participants providing samples at combinations of visits. X-axis: visit combinations; y-axis: intersection size (participants). White plus signs mark intersection dots. Examples: 7 participants provided sputum only at baseline; 2 provided sputum at baseline and Visit 4. For stool, 8 participants contributed samples at all visits; for throat, only 3 did. Left-side bar plots show total samples per visit (set size). Visit colors: baseline = gray, 3 months = yellow, 6–12 months = blue, 15–18 months = red, 21–24 months = green.

e, 16S rRNA gene copy number (qPCR) per sample type and time point. Throat and sputum did not differ, but stool had higher copy numbers (higher bacterial burden; LME: Intercept Sputum 2.606×10^7; Estimate Stool 1.05×10^{9}; Std. Error 1.11×10^{8}; $p < 2\times10^{-16}$). No change over time. Due to funding limits, qPCR was performed only up to 18 months post-ETI, totaling 313 samples.

f, Library size (total reads post-quality control) by sample type and months from ETI start or in healthy controls. No significant difference between months post-ETI and healthy controls, but stool samples had significantly ($p < 0.05$) larger library sizes than sputum or throat.

Box plots show the median (line), IQR (box), 1.5× IQR range (whiskers), and outliers (points beyond whiskers).

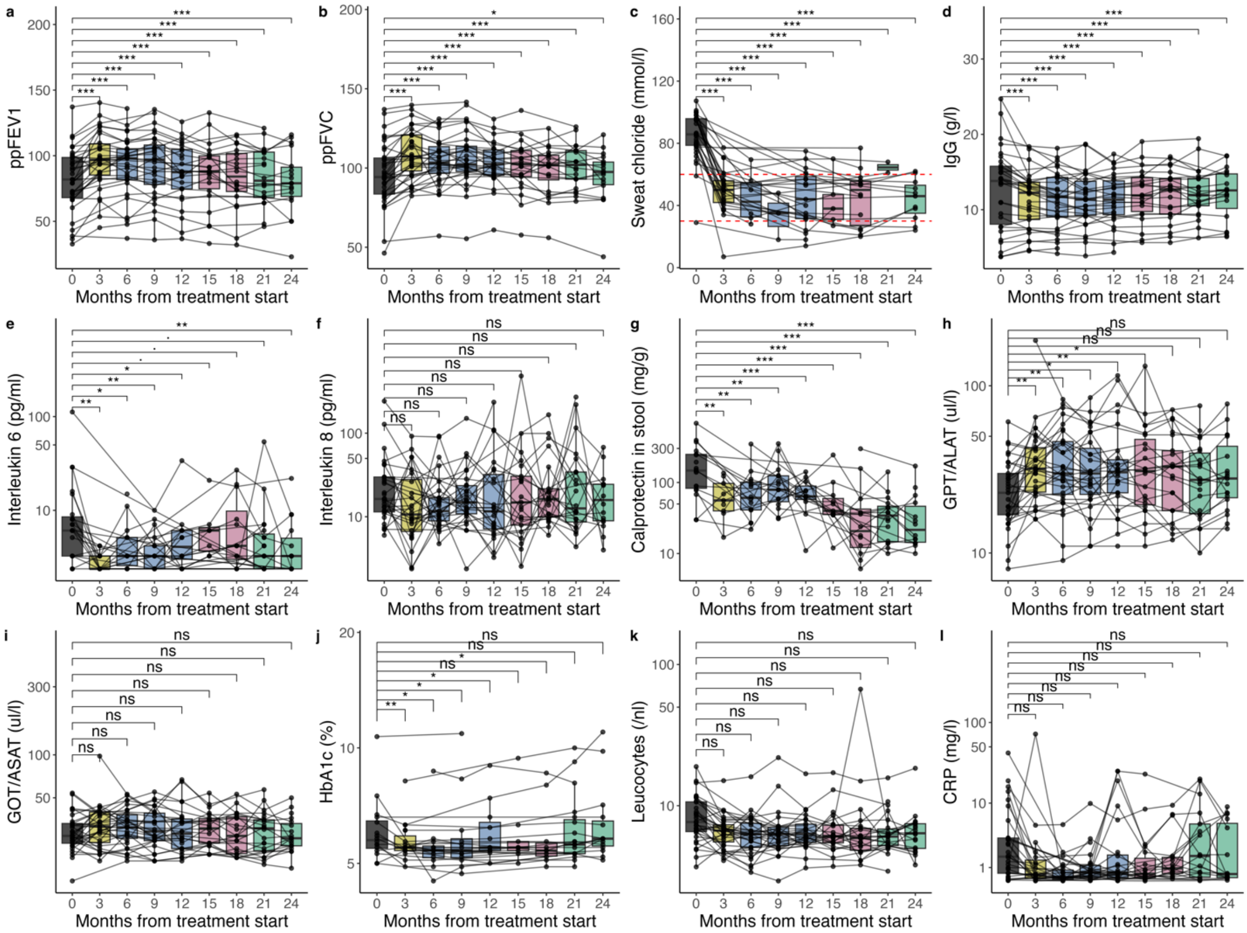

Supplementary Fig. 2:

a-l, **Longitudinal evolution of clinical parameters.** Box plots show the median (line), IQR (box), 1.5× IQR range (whiskers). Points represent individual samples, lines connect intra-individual follow-ups to highlight trajectories, asterisks indicate significance in linear mixed effects models with id as a random factor, corrected for sex and age (FDR***<=0.001, FDR**<=0.01, FDR*<=0.05, FDR$^{.}$<=0.1, FDR^{ns}>=0.1). Colors indicate months from ETI treatment start: baseline=black, 3 months=yellow, 6-12 months=blue, 15-18 months=red, 21-24 months=green. The corresponding N of observations per sample time point and LME results are available in Supplementary Data 2.

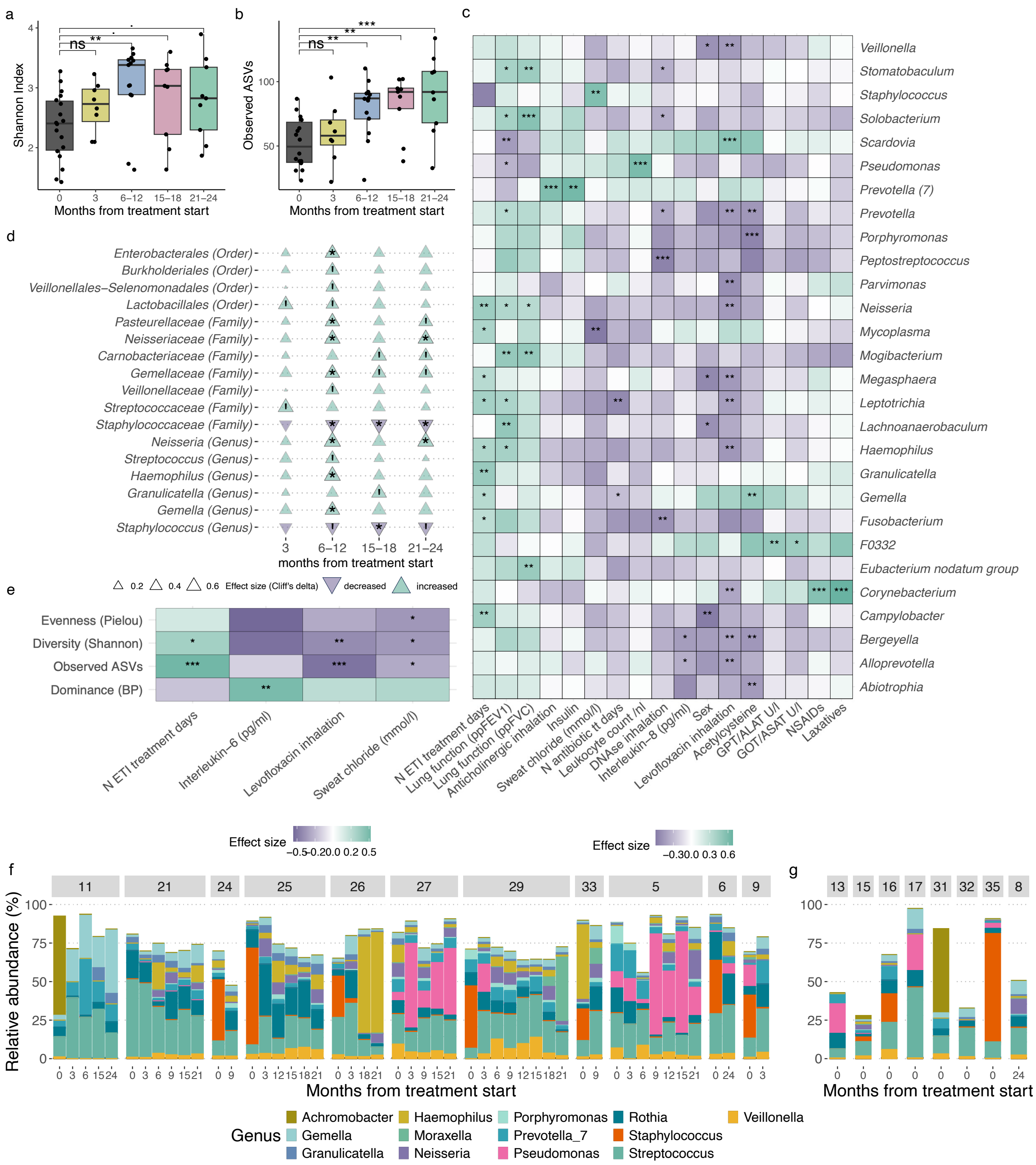

Supplementary Fig. 3: Sputum microbiome taxonomic composition and changes in alpha diversity in response to treatment and other clinical metadata.
a-b, Alpha diversity progression following ETI treatment start for sputum samples, revealing an increase in Shannon Index diversity index (**a**) and the number of observed ASVs per sample (**b**). Box plots show the median (line), IQR (box), 1.5× IQR range (whiskers), and outliers (points beyond whiskers).
c, Genera in sputum significantly associated with clinical metadata. Heatmap hues represent signed effect sizes (Spearman's rho or Cliff's delta), asterisks mark post-hoc univariate significance (FDR ***≤0.001, **≤0.01, *≤0.05). Only genera with ≥1 absolute effect size >0.45 are shown. Full results (including confounded/deconfounded analyses) in Supplementary Data 5. tt = treatment.
d, Reduction in *Staphylococcus* and *Staphylococcaceae* relative abundances observed from 6 months post the initiation of ETI treatment in sputum samples. Cuneiform plot shows significantly altered taxa between baseline and follow-ups (months after ETI start). Marker direction/color = effect direction; marker size = absolute effect size. Solid borders/asterisks = significance (FDR *≤0.05, '≤0.1). See Supplementary Data 7 for details.
e, Alpha diversity metrics (based on rarefied reads to minimum depth) significantly linked to clinical metadata. Heatmap hues = signed effect sizes; asterisks as in (c). Only features with absolute effect sizes >0.2 are displayed. Full statistics in Supplementary Data 8.

f-g, Genus-level composition in sputum samples for genera with >40% prevalence across all samples. Samples grouped by participant ID (gray headers), arranged by collection time point (x-axis). Y-axis = relative abundance (%). Bars = stacked genera composition; colors = genera (legend at right).
f, Participants with multiple sputum samples during the 24-month period.
g, Participants with only one sputum sample at a single time point.

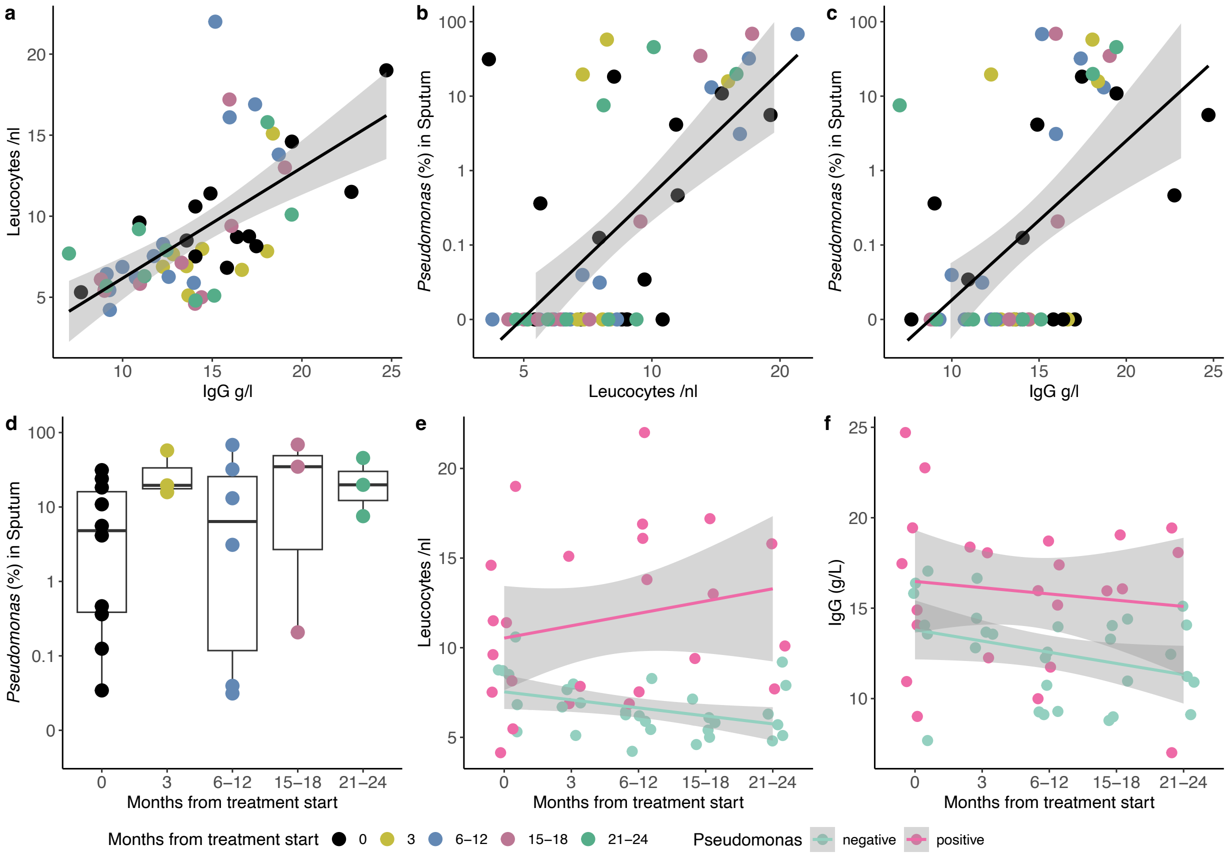

Supplementary Fig. 4: Decrease in serum leukocyte counts depends on *Pseudomonas* detection status in sputum.
We explored the association between *Pseudomonas* relative abundance in sputum and leukocyte count (per nl) and Immunoglobulin G (IgG), in the subgroup of participants with available sputum and serum samples (b-c). Panel (d) focuses on sputum samples positive for *Pseudomonas*, while panels (e) and (f) compare inflammatory markers between *Pseudomonas*-positive (N=25 from 12 participants) and -negative sputum groups (N=32 from 10 participants). For IgG, both groups showed a significant reduction across sampling time points compared to baseline. In the *Pseudomonas*-positive group, the estimated decreases to baseline were: -3.8 (3 months), -5.0 (6-12 months), -5.13 (15-18 months), and -3.5 (21-24 months) (LME, $p < 0.01$). In the *Pseudomonas*-negative group, estimates were: -1.9, -2.4, -2.9, and -2.45 (LME, $p < 0.03$). For leukocytes, significant reductions over time were observed only in the *Pseudomonas*-negative group (LME estimates: -1.6, -2.1, -2.7, and -1.9; $p < 0.05$). In the *Pseudomonas*-positive group, changes in leukocyte count over time were not significant. These findings highlight distinct inflammatory dynamics in relation to *Pseudomonas* colonization status. Box plots show the median (line), IQR (box), 1.5× IQR range (whiskers), and outliers (points beyond whiskers).

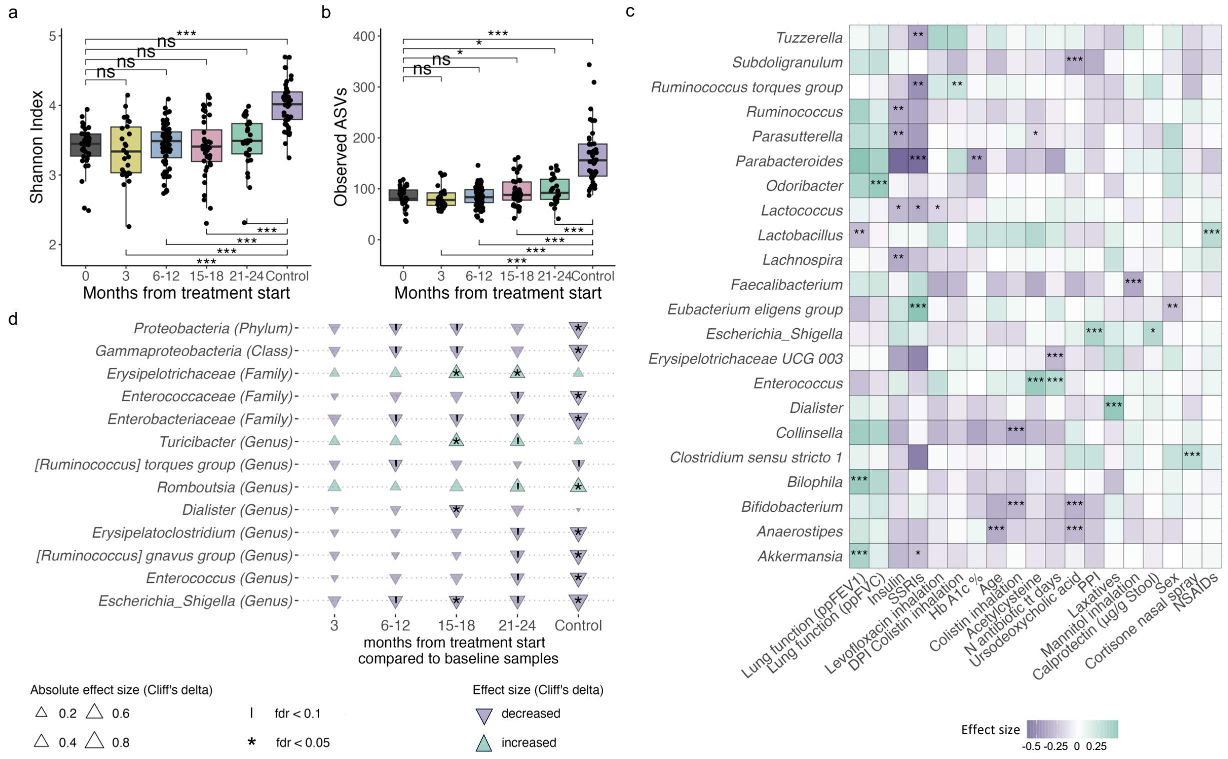

Supplementary Fig. 5: Stool alpha diversity metrics and taxonomic changes in comparison to baseline across sampling timepoints.

a-b, Alpha diversity progression following ETI treatment start for stool samples, revealing no difference in Shannon Index (a) but an increase in the number of observed ASVs per sample (b). Healthy control samples displayed higher alpha diversity metrics (p<0.001) when compared to CF baseline. Box plots show the median (line), IQR (box), 1.5× IQR range (whiskers), and outliers (points beyond whiskers).

c, Genera in stool significantly associated with clinical metadata. Heatmap hues represent signed effect sizes (Spearman's rho or Cliff's delta), with asterisks indicating post-hoc univariate significance (FDR***<=0.001, FDR**<=0.01, FDR*<=0.05). Fig. shows all significant genera with at least one absolute effect size >0.3. Statistical report for significant confounded and deconfounded associations is given in Supplementary Data 5, tt=treatment.

d, Taxonomic changes across sampling timepoints (months after ETI treatment start) in comparison to baseline. In addition control samples are compared to CF baseline samples, with marker direction and color hue indicating direction of effect, and marker size representing absolute effect size. Solid borders and asterisks denote significance (* FDR < 0.05, ' FDR < 0.1). Effect sizes and significance documented in Supplementary Data 7.

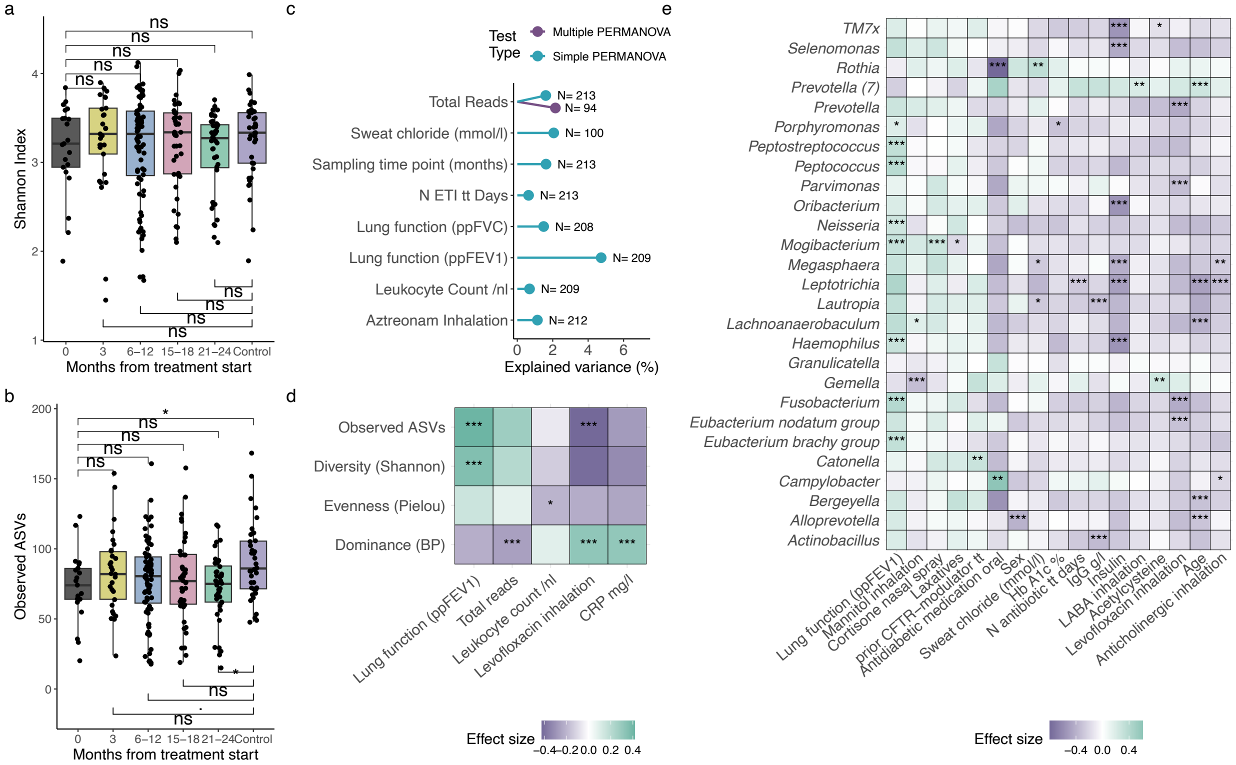

Supplementary Fig. 6: Throat microbiome dynamics across sampling time points and in regard to clinical metadata.

a, Shannon index of throat samples across sampling time points and healthy controls displayed.

b, Number of observed ASVs in throat samples across sampling time points. Healthy controls displayed marginally higher numbers of observed ASVs when compared to CF baseline (FDR = 0.1). Statistical report on performed LMEs is given in Supplementary Data 4, 9. Box plots show the median (line), IQR (box), 1.5× IQR range (whiskers), and outliers (points beyond whiskers).

c, Lollipops are illustrating the variance (R^2 in %) in microbial community composition explained by individual covariates, assessed using BC dissimilarities. Variables that were significant ($p<0.05$) in Simple PERMANOVA are depicted in blue, while variables that retained significance ($p<0.05$) in Multiple PERMANOVA are depicted in purple. Full statistical results are given in Supplementary Data 18.

d, Alpha diversity metrics (calculated on rarefied reads to minimum sequencing depth) significantly associated with clinical metadata. Heatmap hues represent signed effect sizes (Spearman's rho or Cliff's delta),, with asterisks indicating post-hoc univariate significance (FDR***<=0.001, FDR**<=0.01, FDR*<=0.05). Features displayed have absolute effect sizes >0.2. Statistical report for significant confounded and deconfounded associations is given in Supplementary Data 8.

e, Genera in the throat significantly associated with clinical metadata. Heatmap hues represent signed effect sizes (Spearman's rho or Cliff's delta), with asterisks indicating post-hoc univariate significance (FDR***<=0.001, FDR**<=0.01, FDR*<=0.05). Fig. shows all significant genera with at least one absolute effect sizes >0.3. Statistical report for significant confounded and deconfounded associations is given in Supplementary Data 5, tt=treatment, LABA= long-acting beta agonist.

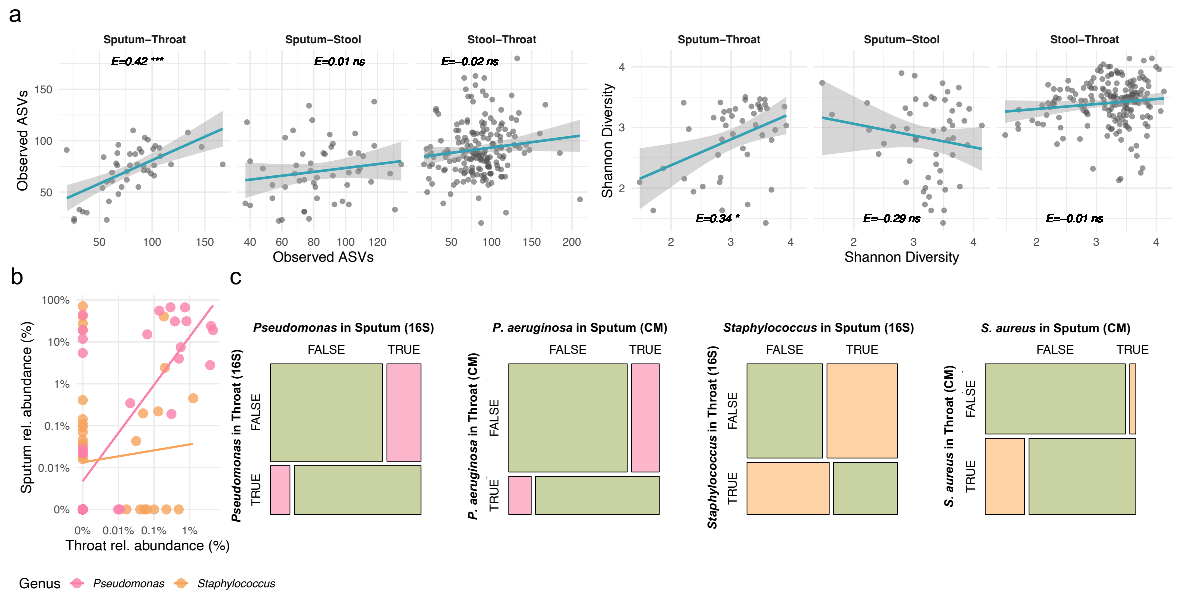

Supplementary Fig. 7: Correlation of alpha-diversity measures between habitats and co-correlation and occurence of *Staphylococcus* and *Pseudomonas* in airway samples

a, Correlation of observed ASVs and Shannon diversity within paired samples between habitats. Line shows linear fit; shaded areas indicate 95% confidence intervals. Significant associations were only observed for sputum-throat comparisons (LME with ID as random effect). No significant relationships were found in gut-respiratory pairs.

b, Relative abundances of *Staphylococcus* and *Pseudomonas* in paired throat and sputum samples. Despite low correlation coefficients (Spearman $\rho < 0.04$), visual trends suggest some samples display coordinated abundance. Lines show linear fit per genus, indicated by color, pink (*Pseudomonas*) and orange (*Staphylococcus*).

c, Mosaic plots showing concordance of presence/absence detection for *Staphylococcus* and *Pseudomonas* in paired throat and sputum samples. Green cells denote true positives/negatives; pink (*Pseudomonas*) and orange (*Staphylococcus*) cells represent false positives/negatives. Detection of *Pseudomonas* with 16S rRNA gene sequencing (16S) in throat samples showed sensitivity of 87% and specificity of 77% for sputum presence. Accuracy between the 2 sample sites was 80%, more details in Supplementary Data 15.

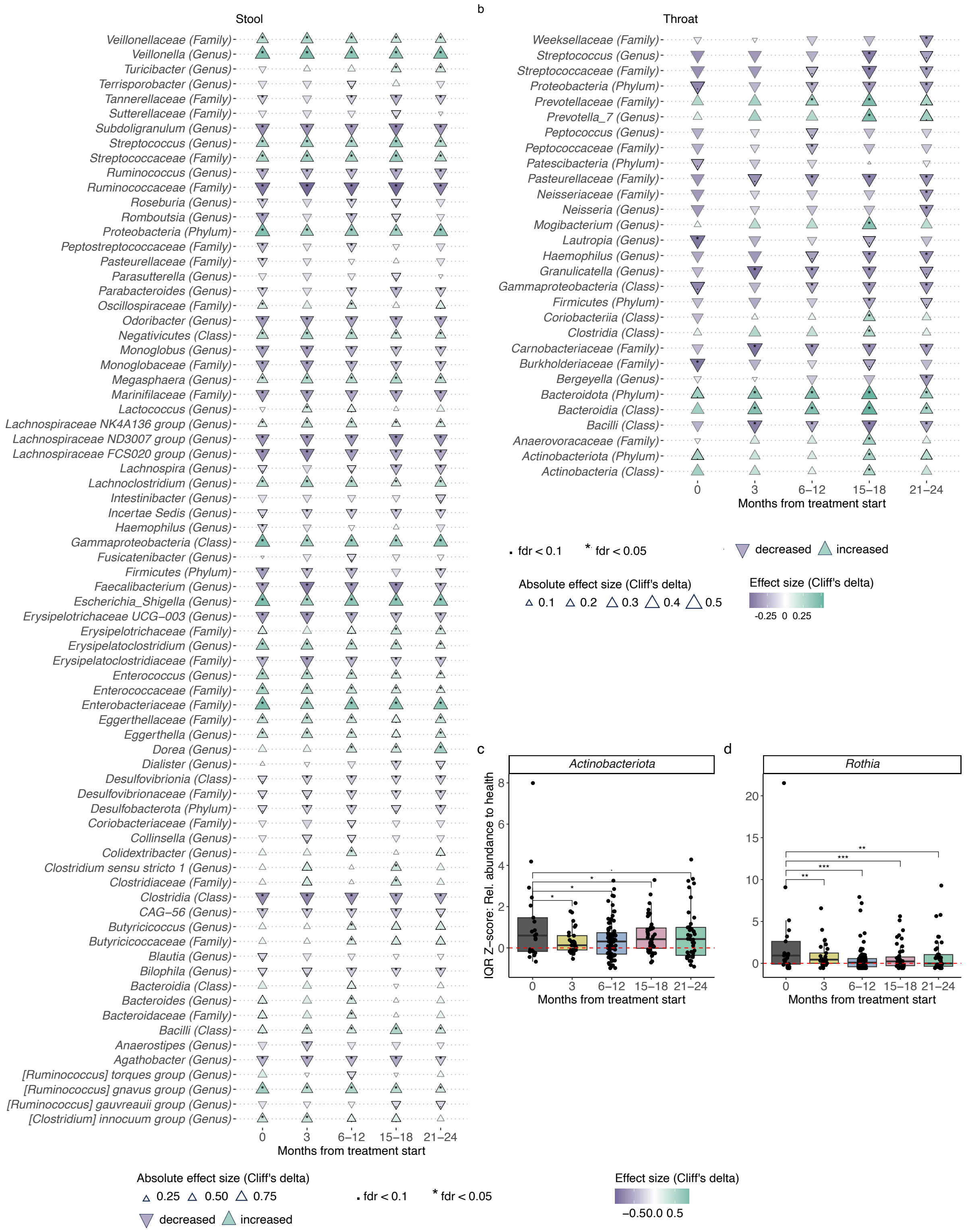

Supplementary Fig. 8: Taxonomic differential abundances between healthy control samples and CF samples across time points.

a-b, Significantly altered bacterial taxa at various taxonomic levels in stool (**a**) and in throat (**b**) between healthy control samples compared to each CF sample timepoint (months after ETI treatment start), with marker 6direction and color hue indicating direction of effect, and color hue and marker size representing absolute effect size. Solid borders (FDR<0.1) and asterisks denote significance (FDR < 0.1 (.), FDR < 0.05 (*), FDR < 0.01 (**), and FDR < 0.001 (***).

c-d, Taxa for which IQR-Z Score changes over ETI treatment time in CF throat samples and align towards abundances observed in healthy controls. Boxplots depict the distribution of IQR-Z scores for microbiome taxa across different time points following ETI treatment initiation (Baseline, 3 months, 6-12 months, etc.). Z-score of 0 indicates no deviation from the control median, and is depicted as a dashed red horizontal line. Individual dots

overlaid on the boxplots represent individual samples, showing the spread and variation in Z-scores across the time points. Significance of changes compared to baseline (0 months) is determined using a linear mixed-effects model (lme), adjusted for participant ID as a random factor. Statistically significant differences are indicated by asterisks, with FDR-corrected p-values denoted as follows: FDR < 0.1 (.), FDR < 0.05 (*), FDR < 0.01 (**), and FDR < 0.001 (***).

2. Supplementary Tables:

Supplementary Table S1: CF microbiome and ETI studies

Ref.	First author (year)	Study design & follow-up	Sample type(s)	Has healthy controls	Notes / comparison to our study	Cited
1	Schaupp (2023)	Single center, larger initial cohort but high dropout (n=65 → n=28 at 12 months), 12-month follow-up	Sputum	Yes, (n=10)	*P. aeruginosa* reduction at 3, but not at 12 months, healthy levels not reached, shorter follow-up; larger cohort	✓
2	Martin (2023)	Single center; 7 pwCF on ETI, 9 pwCF no ETI; only 2 individuals followed >1 year	Sputum	No	Very small sample; minimal long-term data	❌
3	Nichols (2023)	Multi-center PROMISE study, 236 pwCF, 6-month follow-up	Sputum	No	PCR and culture methods, no microbiome profiling; most participants remain infected with pathogens	✓
4	Armbruster (2024)	Single center, 16 pwCF, only one post-ETI sample at max 12-month follow-up	Sinus, throat	No	Only one follow-up sample per person; Persistence and evolution of *P. aeruginosa*	✓
5	Hilliam (2024)	Single center, 38 adults but only 21 post-ETI participants, 12-months max follow-up	Sinus, Sputum	No	Interesting sinus dynamics; *P. aeruginosa* and *S.aureus* persist in sinuses; limited long-term data	❌
6	Sosinski (2022)	24 adults, one single sample after early ETI initiation	Sputum	No	Small data set, first study in the field	✓
7	Zemke (2024)	23 adults; one follow-up at median 9 months post-ETI	Sinus	No	No change in *Staphylococcus;* limited longitudinal data	❌
8	Steinberg (2025)	20 adolescents, 3-month follow-up	Throat	No	Very short follow-up	❌
9	Pallenberg (2022)	Single center, 31 pwCF, 11-month follow-up	Throat, (Sputum at baseline)	No	Reduction of *S. aureus* and *P. aeruginosa*	✓
10	Duong (2025)	Multi-center PROMISE study; 124 participants; 1 and 6-month post-ETI stool samples	Stool	No	Large cohort but limited follow-up; supports our *E. coli* and calprotectin data	❌* *very recently published

11	Marsh (2024)	20 pwCF; samples at 3, 6, and >17 months of ETI treatment	Stool	Yes, (n=10)	Moderate follow-up; smaller sample size	✓

References:

1. Schaupp, L. *et al.* Longitudinal effects of elexacaftor/tezacaftor/ivacaftor on sputum viscoelastic properties, airway infection and inflammation in patients with cystic fibrosis. *Eur Respir J* **62**, (2023).
2. Martin, C. *et al.* Longitudinal microbial and molecular dynamics in the cystic fibrosis lung after Elexacaftor–Tezacaftor–Ivacaftor therapy. *Respir Res* **24**, 1–14 (2023).
3. Nichols, D. P. *et al.* Pharmacologic improvement of CFTR function rapidly decreases sputum pathogen density, but lung infections generally persist. *Journal of Clinical Investigation* **133**, (2023).
4. Armbruster, C. R. *et al.* Persistence and evolution of Pseudomonas aeruginosa following initiation of highly effective modulator therapy in cystic fibrosis. *mBio* **15**, 1–8 (2024).
5. Hilliam, Y. *et al.* Following Initiation of Elexacaftor / Tezacaftor / Ivacaftor Therapy. **12**, 1–18 (2024).
6. Sosinski, L. M. *et al.* A restructuring of microbiome niche space is associated with Elexacaftor-Tezacaftor-Ivacaftor therapy in the cystic fibrosis lung. *Journal of Cystic Fibrosis* (2021) doi:10.1016/j.jcf.2021.11.003.
7. Zemke, A. C. *et al.* Elexacaftor–tezacaftor–ivacaftor decreases pseudomonas abundance in the sinonasal microbiome in cystic fibrosis. *International Forum of Allergy and Rhinology* vol. 14 928–938 Preprint at https://doi.org/10.1002/alr.23288 (2024).
8. Steinberg, R. *et al.* Longitudinal effects of elexacaftor/tezacaftor/ivacaftor on the oropharyngeal metagenome in adolescents with cystic fibrosis. *Journal of Cystic Fibrosis* **24**, 562–570 (2025).
9. Pallenberg, S. T. *et al.* Impact of Elexacaftor/Tezacaftor/Ivacaftor Therapy on the Cystic Fibrosis Airway Microbial Metagenome. *Microbiol Spectr* **10**, e0145422 (2022).
10. Duong, J. T. *et al.* Fecal microbiota changes in people with cystic fibrosis after 6 months of elexacaftor/tezacaftor/ivacaftor: Findings from the promise study. *Journal of Cystic Fibrosis* **24**, 792–800 (2025).
11. Marsh, R. *et al.* Impact of extended Elexacaftor/Tezacaftor/Ivacaftor therapy on the gut microbiome in cystic fibrosis. *Journal of Cystic Fibrosis* **23**, 967–976 (2024).
