# Supplementary material for: CFTR modulator therapy drives microbiome restructuring through improved host physiology in cystic fibrosis: the IMMProveCF phase IV trial
Source: Nat Commun. 2025 Nov 18;16:10111. doi: 10.1038/s41467-025-64218-z (PMC12627475; doi:10.1038/s41467-025-64218-z)
Supplement: Supplementary file 2 — Description of Additional Supplementary Files [file 41467_2025_64218_MOESM2_ESM.pdf]

Supplementary Data legends:

File Name: Supplementary Data 1: Linear mixed effect model results on the delta of clinical parameters.
Description: This table presents the results of linear mixed-effect model results on the change (delta) in clinical parameters, representing the difference between baseline and follow-up measurements as depicted in Fig. 1b-e: Dependent variable ~ Variable + Sex + Age + (1 | ID), with p-values corrected by the false discovery rate (FDR). "Variable"(= predictor) denotes the number of months after ETI treatment initiation, with the baseline timepoint as the intercept. Estimates represent changes from baseline: positive values indicate an increase, and negative values indicate a decrease in the dependent variable. Each estimate shows the change in the outcome for a one-unit change in the predictor, controlling for age and sex. Age and sex are included as fixed effects, with their independent estimates on the outcome variable provided. The term (1 | ID) accounts for repeated measures within individuals by including a random intercept for each participant, which adjusts for individual differences and allows the model to handle the non-independence of repeated measurements from the same individual.The table also includes N_obs, the number of observations per time point, and Total_N_obs, the total number of observations for the dependent variable.

File Name: Supplementary Data 2: Linear mixed effects model results of all clinical parameters.
Description: This table presents the results of Linear mixed effects models on the total values of clinical parameters as depicted in SupplementaryFig. 2. The same LME formula, abbreviations and explanations as for Supplementary Data 1.

File Name: Supplementary Data 3: Multiple PERMANOVA results per sample type for variance explained by time point of sampling.
Description: This table presents the results of the PERMANOVA on beta-diversity (BC dissimilarities) per sample type, stratified by participant ID. The PERMANOVA examines the combined influence of sample time point (visit_sum), sex and age (in years) of the participant on the variance in microbiome composition. **Df (Degrees of Freedom):** The number of independent values or quantities that can vary for each factor. **SumOfSqs (Sum of Squares):** The total variation attributed to each factor. **R2:** The proportion of variance explained by each factor. **F:** The F-statistic from the PERMANOVA, indicating the ratio of variance explained by the factor to the unexplained variance. **Pr(>F) (p-value):** The significance level of the factor, derived from 999 permutations. **Signif.:** Asterixis denote the significance level of the p-value: '***'< 0.001; '**'<0.01; '*'<0.05; '.' < 0.1.

File Name: Supplementary Data 4: Linear mixed effects model results for alpha diversity estimates.
Description: This table presents the results of Linear mixed effects models on alpha diversity estimates (Shannon Index, richness=observed ASVs, evenness= Pielou Index, dominance=Berger Parker Index) between baseline samples and follow-up samples. We show alpha diversity measures calculated on rarefied and unrarefied (raw) read counts. LME-formula: Alpha diversity estimate ~ Variable + Sex + Age + (1 | ID). Variable"(= predictor) denotes the number of months after ETI treatment initiation, with the baseline timepoint as the intercept. P-values are corrected by the false discovery rate (FDR). Estimates represent changes from baseline: positive values indicate an increase, and negative values indicate a decrease in the dependent variable. Each estimate shows the change in the outcome for a one-unit change in the predictor, controlling for age and sex. Age and sex are included as fixed effects, with their independent estimates on the outcome variable provided. The term (1 | ID) accounts for repeated measures within individuals by including a random intercept for each participant, which adjusts for individual differences and allows the model to handle the non-independence of repeated measurements from the same individual.

File Name: Supplementary Data 5: Statistical report for significant confounded and deconfounded associations between clinical and technical parameters and genera for sputum, throat and stool samples.
Description: Results are sorted by sample type. It includes raw p-values (Ps), multiple testing corrected p-values (Qs), effect sizes (Ds), and confounding status (status) for each combination of a tested genus (feature) and a meta-variable (full list in Supplementary Data 6). **Ps, Qs, and Ds** are based on naive association tests: wilcox.test() for binary, cor.test() for continuous numerical, and kruskal.test() for neither numeric nor binary variables. **Status labels** reflect the effects of included random/fixed effects in confounder modeling and post hoc testing: **OK_nc**: No covariate, trivially deconfounded results; **OK_sd**: significantly deconfounded, valid associations not reducible to other covariates; **C:** Followed by meta-variables, indicating which meta-variables confound the association; **AD**: Ambiguously deconfounded results where both the tested meta-variable and covariate signals are lost. We included participant ID as a random factor to control for repeated measures.

File Name: Supplementary Data 6: Tested Variables.
Description: This table lists the 58 clinical and technical variables we used in our covariate aware analysis. We give explanations on variables and abbreviations.

File Name: Supplementary Data 7: Effect sizes and significance of taxonomic alterations in comparison to baseline across sample types.

Description: This table presents the results of the analysis of taxonomic alterations relative to the baseline samples (V1) across different time points, ordered by sample type. Each row represents a comparison of taxonomic changes between the baseline (V1) and subsequent time points (V2=3 months, V3V5=6-12 months, V6V7=15-18 months, V8V9= 21-24 months). The analysis was conducted using a Wilcoxon signed-rank test, and differences were further corrected for repeated measures by including the participant ID as a random factor in a post hoc linear model. **status**: The significance status of the comparison, where "NS" denotes not significant, "OK_nc" denotes significant with no covariate, and other statuses denote different levels of significance with respective confounder effects. **raw_p**: The raw p-value from the initial Wilcoxon signed-rank test. **comparison_p**: The time points being compared, with V1 as the baseline. **effectSize**: The effect size, representing the magnitude of taxonomic change from the baseline. Negative values indicate a decrease. **corr_p**: The p-value corrected for multiple testing via FDR. **FDR**: The false discovery rate adjusted p-value denoted as * FDR < 0.05, . FDR < 0.1, ns denotes not significant. **taxa annotation**: The taxonomic group analyzed, e.g. *Staphylococcus*.

File Name: Supplementary Data 8: Results of covariate aware analysis with MetadeconfoundR on diversity estimates.
Description: Explanations are equivalent to those presented in Supplementary Data 5.

File Name: Supplementary Data 9: Linear mixed effects model results for alpha diversity estimates CF sampling time-points compared to Control.
Description: This data table presents the results of Linear mixed effects models on alpha diversity estimates (Shannon Index, richness=observed ASVs, evenness= Pielou Index, dominance=Berger Parker Index) between Control samples and CF samples, where Control samples are representing the intercept that all CF samples are compared to according to their sample time point. Here we show results on rarefied alpha diversity measures only. LME formula and explanations are equivalent to LMEs presented in Supplementary Data 4.

File Name: Supplementary Data 10: Effect sizes and significance of taxonomic alterations in comparison to Control across sample types.
Description: This table presents the results of the analysis of taxonomic alterations relative to the healthy control samples (Control) across different time points, ordered by sample type. Explanations are equivalent to those presented in Supplementary Data 7.

File Name: Supplementary Data 11: Linear mixed effects model results for Z-scores across sampling time points in comparison to baseline.
Description: P-values are corrected by the false discovery rate (FDR). Estimates represent changes from baseline: positive values indicate an increase, and negative values indicate a decrease in the Z-Score. Each estimate shows the change in the outcome for a one-unit change in the predictor, controlling for age and sex. Age and sex are included as fixed effects, with their independent estimates on the outcome variable provided.The term (1 | ID) accounts for repeated measures within individuals by including a random intercept for each participant, which adjusts for individual differences and allows the model to handle the non-independence of repeated measurements from the same individual.

File Name: Supplementary Data 12: Overview of all screened bacterial lung and gut microbiome members.
Description: This lists all bacterial strains from lung and gut microbiomes that were screened in this study, including their source, growth medium and culture conditions, and relevant references.

File Name: Supplementary Data 13: Drugs and solvent included in the screen.
Description: This provides the manufacturers of the drugs and solvent included in the screening assays.

File Name: Supplementary Data 14: Alpha diversity indices on unrarefied and rarefied counts.
Description: This table presents the diversity indices calculated on both unrarefied and rarefied counts for various alpha diversity measures. The alpha diversity indices included in this analysis are the Shannon Index, richness (observed ASVs), evenness (Pielou Index), and dominance (Berger Parker Index). The corresponding values are displayed for each alpha diversity index, including the mean and standard deviation (SD), as well as the median with the minimum and maximum values. The calculation of these indices was performed using the alpha function of the microbiome package. The analysis was conducted on both rarefied and unrarefied (raw) read counts across different sampling time points and for the Control samples. The table also provides information on the number of samples (N) for each sample time point and sample type (sputum, throat, stool).

File Name: Supplementary Data 15: Contingency table for Pseudomonas and Staphylococcus detection across comparisons of sample types (sputum vs. throat) and methods (culture (CM) vs. 16S rRNA gene sequencing).
Description: In each comparison, either CM or sputum is used as the reference (gold standard), hence for CM vs. 16S: FN = CM-positive but 16S-negative; FP = CM-negative but 16S-positive. For Sputum vs. Throat: FN = sputum-positive but throat-negative; FP = sputum-negative but throat-positive. CM= Conventional Microbiology (Culture), 16S= 16S rRNA gene sequencing, N= Number of paired samples, TN=True Negatives, FP = False

Positives, FN= False Negatives, and TP=True Positives. Accuracy= TP+TN/N; Sensitivity = TP / (TP + FN); Specificity = TN / (TN + FP).

File Name: Supplementary Data 16: Simple PERMANOVA stratified by sample donor ID.
Description: This table presents the results of the PERMANOVA on beta-diversity (BC dissimilarities) per sample type, stratified by participant ID on combined and stand-alone sample type data sets, as depicted in Fig. 5 a+b.

File Name: Supplementary Data 17: PERMANOVA on BC-dissimilarities between sampling time points, stratified by donor (not for control comparisons).
Description: This table summarizes the PERMANOVA results on beta-diversity (Bray-Curtis dissimilarities) per sample type, with analyses stratified by participant ID. Time points are defined as follows: 1 = baseline, 2 = 3 months after ETI treatment start, 3–5 = 6–12 months, 6–7 = 15–18 months, and 8–10 = 21–24 months, control = healthy control samples. Corresponding visualizations are shown in Figures 2b, 3b, 4b, and 6a.

File Name: Supplementary Data 18: Simple and multiple PERMANOVA results stratified by donor.
Description: This table presents the variance in microbial composition (Bray-Curtis dissimilarities) explained by individual covariates, assessed through both simple and multiple PERMANOVA models. Significant variables ($p < 0.05$) are highlighted: blue indicates significance in simple models, and purple in multiple models, as depicted in the lollipop plots. Visualizations of these results are provided in Figures 2d, 3d, and SupplementaryFigure 6c.
