# Supplementary material for: CFTR modulator therapy drives microbiome restructuring through improved host physiology in cystic fibrosis: the IMMProveCF phase IV trial
Source: Nat Commun. 2025 Nov 18;16:10111. doi: 10.1038/s41467-025-64218-z (PMC12627475; doi:10.1038/s41467-025-64218-z)
Supplement: Supplementary file 4 — Reporting Summary [file 41467_2025_64218_MOESM4_ESM.pdf]

nature portfolio

Corresponding author(s): Rebecca L. Knoll, Sofia K. Forslund-Startceva

Last updated by author(s): Aug 14, 2025

Reporting Summary

Nature Portfolio wishes to improve the reproducibility of the work that we publish. This form provides structure for consistency and transparency in reporting. For further information on Nature Portfolio policies, see our Editorial Policies and the Editorial Policy Checklist.

Statistics

For all statistical analyses, confirm that the following items are present in the figure legend, table legend, main text, or Methods section.

n/a	Confirmed	
☐	☒	The exact sample size (*n*) for each experimental group/condition, given as a discrete number and unit of measurement
☐	☒	A statement on whether measurements were taken from distinct samples or whether the same sample was measured repeatedly
☐	☒	The statistical test(s) used AND whether they are one- or two-sided<br>*Only common tests should be described solely by name; describe more complex techniques in the Methods section.*
☐	☒	A description of all covariates tested
☐	☒	A description of any assumptions or corrections, such as tests of normality and adjustment for multiple comparisons
☐	☒	A full description of the statistical parameters including central tendency (e.g. means) or other basic estimates (e.g. regression coefficient) AND variation (e.g. standard deviation) or associated estimates of uncertainty (e.g. confidence intervals)
☐	☒	For null hypothesis testing, the test statistic (e.g. *F*, *t*, *r*) with confidence intervals, effect sizes, degrees of freedom and *P* value noted<br>*Give P values as exact values whenever suitable.*
☒	☐	For Bayesian analysis, information on the choice of priors and Markov chain Monte Carlo settings
☐	☒	For hierarchical and complex designs, identification of the appropriate level for tests and full reporting of outcomes
☐	☒	Estimates of effect sizes (e.g. Cohen's *d*, Pearson's *r*), indicating how they were calculated

Our web collection on statistics for biologists contains articles on many of the points above.

Software and code

Policy information about availability of computer code

Data collection	No code was used to collect data, data was collected by the clinical study team into Microsoft® Excel spreadsheets.
Data analysis	All analysis scripts are available at https://github.com/RebeccaLuise/IMMProveCF_public. 10.5281/zenodo.16878229. All analysis was carried out in R (v4.1). In the following we list the imported R packages, followed by the version employed, to facilitate the analysis:<br>"dada2","1.22.0"<br>"mediation","4.5.0"<br>"ggpubr","0.4.0"<br>"rockchalk","1.8.157"<br>"multilevel","2.7"<br>"bda","18.3.2"<br>"gvlma","1.0.0.3"<br>"stargazer","5.2.3"<br>"dbplyr","2.5.0"<br>"factoextra","1.0.7"<br>"ggalluvial","0.12.5"<br>"ggplot2","3.5.1"<br>"tidyverse","2.0.0"<br>"phyloseq","1.38.0"<br>"magrittr","2.0.3"

"janitor","2.1.0"
"microbiome","1.16.0"
"knitr","1.48"
"lubridate","1.9.3"
"naniar","1.1.0"
"stringr","1.5.1"
"ggpubr","0.6.0"
"gtsummary","1.7.2"
"rmarkdown","2.28"
"readxl","1.4.3"
"rstatix","0.7.2"
"metadeconfoundR","1.0.2"
"metacoder","0.3.7"
"agricolae","1.3-5"
"ape","5.8"
"gridExtra","2.3"
"dendextend","1.17.1"
"vegan","2.6-8"
"ComplexUpset","1.3.3"
"microViz","0.9.3"
"paletteer","1.4.0"
"datarium","0.1.0"

For manuscripts utilizing custom algorithms or software that are central to the research but not yet described in published literature, software must be made available to editors and reviewers. We strongly encourage code deposition in a community repository (e.g. GitHub). See the Nature Portfolio guidelines for submitting code & software for further information.

Data

Policy information about availability of data

All manuscripts must include a data availability statement. This statement should provide the following information, where applicable:

- Accession codes, unique identifiers, or web links for publicly available datasets
- A description of any restrictions on data availability
- For clinical datasets or third party data, please ensure that the statement adheres to our policy

All annotated sequencing data and metadata are available at https://github.com/RebeccaLuise/IMMProveCF_public with raw sequences accessible at http://www.ncbi.nlm.nih.gov/bioproject/1080555 (PRJNA1080555).

Research involving human participants, their data, or biological material

Policy information about studies with human participants or human data. See also policy information about sex, gender (identity/presentation), and sexual orientation and race, ethnicity and racism.

Reporting on sex and gender	We collected biological sex as part of our clinical metadata. Since long-term clinical outcomes in cystic fibrosis are known to be influenced by sex— with females generally experiencing more severe disease progression— we accounted for this variable in our analysis. Specifically, in our univariate analysis, we adjusted our linear mixed-effects models for sex to ensure that reported effect sizes and p-values/FDR are controlled for its potential impact.
Reporting on race, ethnicity, or other socially relevant groupings	The population had a Caucasian-European background recruited in Germany.
Population characteristics	Characteristics of the participants with cystic fibrosis (CF) at baseline and the healthy controls were as follows.<br>Sex<br>• N Female: CF Baseline 18 (51%), Controls 29 (59%)<br>• N Male: CF Baseline 17 (49%), Controls 20 (41%)<br>Age in years<br>• Median (IQR): CF Baseline 24 (13, 31), Controls 24 (18, 33)<br>• Min-Max: CF Baseline 6-53, Controls 6-56<br>Mutation<br>• N F508del heterozygous: CF Baseline 9 (26%)<br>• N F508del homozygous: CF Baseline 26 (74%)<br>Lung function<br>• ppFEV1 (Median, IQR): CF Baseline 82 (68, 99)<br>• ppFVC (Median, IQR): CF Baseline 94 (84, 106)<br>Sweat chloride [mmol/l]<br>• Median (IQR): CF Baseline 86 (79, 96)<br>Bacterial culture results<br>Sputum<br>• Staphylococcus aureus positive: CF Baseline 11 (61%)

- Pseudomonas aeruginosa positive: CF Baseline 10 (56%)
- No culture results: CF Baseline 17 (49%)

Throat
- Staphylococcus aureus positive: CF Baseline 17 (71%)
- Pseudomonas aeruginosa positive: Baseline 5 (21%)
- No culture results: CF Baseline 11 (31%)

Recruitment

Participants were recruited from a single cystic fibrosis (CF) outpatient center in Mainz, Germany. Healthy controls were recruited from the local community through flyers and direct outreach.
CF participants may be biased toward individuals who are either particularly affected by their disease or highly interested in disease pathophysiology and the value of research. Similarly, healthy control participants may be biased toward individuals with a general interest in medical research. For minors, these biases may reflect their guardians' interests and socioeconomic background. However, we do not expect these potential biases to have a major impact on our investigated outcome parameters. Additionally, we examined nutrition as a potential confounding variable, as it is likely to reflect socioeconomic background.

Ethics oversight

Approval for the study design was obtained from the local ethics committee (Landesärztekammer Rheinland-Pfalz 2020-15541), and participant recruitment adhered to the principles of the Declaration of Helsinki. Additionally, the study was registered in the German Clinical Trials Register under the identifier DRKS00023862.

Note that full information on the approval of the study protocol must also be provided in the manuscript.

Field-specific reporting

Please select the one below that is the best fit for your research. If you are not sure, read the appropriate sections before making your selection.

[x] Life sciences [ ] Behavioural & social sciences [ ] Ecological, evolutionary & environmental sciences

For a reference copy of the document with all sections, see nature.com/documents/nr-reporting-summary-flat.pdf

Life sciences study design

All studies must disclose on these points even when the disclosure is negative.

Sample size

The number of recruited participants was determined based on feasibility for a single-center study. For reference power calculation, we considered the scenario of testing for a significant difference between two time points using a paired test. With N = 35 CF participants, an alpha threshold of 0.05, and a target of 80% statistical power, we estimated the minimal effect size required to achieve this power using the pwr R package. A paired t-test was used as the closest implemented equivalent to our nonparametric paired tests. The estimated standardized effect size (Cohen's d) is 0.49, which falls within the range of moderate effect sizes commonly reported in microbiome studies. Therefore, our study is comparably powered relative to similar studies given the sample size recruited.

Data exclusions

No participants were excluded after they and if applicable their legal guardians had given written informed consent. All analyses were conducted with the "na.action = exclude" parameter, ensuring that all available data points for each participant were used (e.g., if a participant provided five out of nine possible throat samples across the study, all five were included in the analysis). No imputation of missing values was performed.

Replication

Clinical parameters were assesed by standarized SOPs. Microbiome, drug-bacteria interaction testing was evaluated by standardized and well-documented pipelines. 16S rRNA sequencing samples were processed and analyzed as singlets, while for the in vitro drug-bacteria interaction testing two biological replicates were tested on one plate.

Randomization

As this study was solely observational and non-interventional, there was no randomization into study arms. All participants followed their standard clinical care, which included the introduction of the new ETI treatment after the baseline visit, and data were collected as part of routine follow-up.

Blinding

Blinding was not applicable to this study, as all CF participants underwent ETI treatment following baseline sampling. Additionally, key clinical outcome parameters, such as lung function and sweat chloride, showed distinct differences between baseline and follow-up samples. Given these clear distinctions, blinding would not have been effective in minimizing bias.

Reporting for specific materials, systems and methods

We require information from authors about some types of materials, experimental systems and methods used in many studies. Here, indicate whether each material, system or method listed is relevant to your study. If you are not sure if a list item applies to your research, read the appropriate section before selecting a response.

Materials & experimental systems

n/a	Involved in the study
☐	☐ Antibodies
☐	☐ Eukaryotic cell lines
☐	☐ Palaeontology and archaeology
☐	☐ Animals and other organisms
☐	☒ Clinical data
☐	☐ Dual use research of concern
☐	☐ Plants

Methods

n/a	Involved in the study
☐	☐ ChIP-seq
☐	☐ Flow cytometry
☐	☐ MRI-based neuroimaging

Antibodies

Antibodies used

Describe all antibodies used in the study; as applicable, provide supplier name, catalog number, clone name, and lot number.

Validation

Describe the validation of each primary antibody for the species and application, noting any validation statements on the manufacturer's website, relevant citations, antibody profiles in online databases, or data provided in the manuscript.

Eukaryotic cell lines

Policy information about cell lines and Sex and Gender in Research

Cell line source(s)

State the source of each cell line used and the sex of all primary cell lines and cells derived from human participants or vertebrate models.

Authentication

Describe the authentication procedures for each cell line used OR declare that none of the cell lines used were authenticated.

Mycoplasma contamination

Confirm that all cell lines tested negative for mycoplasma contamination OR describe the results of the testing for mycoplasma contamination OR declare that the cell lines were not tested for mycoplasma contamination.

Commonly misidentified lines
(See ICLAC register)

Name any commonly misidentified cell lines used in the study and provide a rationale for their use.

Palaeontology and Archaeology

Specimen provenance

Provide provenance information for specimens and describe permits that were obtained for the work (including the name of the issuing authority, the date of issue, and any identifying information). Permits should encompass collection and, where applicable, export.

Specimen deposition

Indicate where the specimens have been deposited to permit free access by other researchers.

Dating methods

If new dates are provided, describe how they were obtained (e.g. collection, storage, sample pretreatment and measurement), where they were obtained (i.e. lab name), the calibration program and the protocol for quality assurance OR state that no new dates are provided.

☐ Tick this box to confirm that the raw and calibrated dates are available in the paper or in Supplementary Information.

Ethics oversight

Identify the organization(s) that approved or provided guidance on the study protocol, OR state that no ethical approval or guidance was required and explain why not.

Note that full information on the approval of the study protocol must also be provided in the manuscript.

Animals and other research organisms

Policy information about studies involving animals; ARRIVE guidelines recommended for reporting animal research, and Sex and Gender in Research

Laboratory animals

For laboratory animals, report species, strain and age OR state that the study did not involve laboratory animals.

Wild animals

Provide details on animals observed in or captured in the field; report species and age where possible. Describe how animals were caught and transported and what happened to captive animals after the study (if killed, explain why and describe method; if released, say where and when) OR state that the study did not involve wild animals.

Reporting on sex

Indicate if findings apply to only one sex; describe whether sex was considered in study design, methods used for assigning sex. Provide data disaggregated for sex where this information has been collected in the source data as appropriate; provide overall numbers in this Reporting Summary. Please state if this information has not been collected. Report sex-based analyses where performed, justify

	reasons for lack of sex-based analysis.
Field-collected samples	*For laboratory work with field-collected samples, describe all relevant parameters such as housing, maintenance, temperature, photoperiod and end-of-experiment protocol OR state that the study did not involve samples collected from the field.*
Ethics oversight	*Identify the organization(s) that approved or provided guidance on the study protocol, OR state that no ethical approval or guidance was required and explain why not.*

Note that full information on the approval of the study protocol must also be provided in the manuscript.

Clinical data

Policy information about clinical studies

All manuscripts should comply with the ICMJEguidelines for publication of clinical research and a completedCONSORT checklist must be included with all submissions.

Clinical trial registration	DRKS00023862 (German Clinical Trials Register)
Study protocol	The full protocol is in English language and it can be requested at rebknoll@uni-mainz.de
Data collection	The first patient was enrolled in October 2020, and the last patient sample to be included in the analysis was in June 2023.
Outcomes	Primary outcomes were longitudinal changes in diversity, species richness, and microbial composition of the respiratory and intestinal microbiome during treatment with the triple CFTR-modulator therapy (elexacaftor/tezacaftor/ivacaftor), assessed via 16S rRNA gene sequencing.<br>Secondary outcomes included:<br>• Changes in sweat chloride concentration as a proxy for CFTR function.<br>• Lung function assessment using spirometry to measure changes in ppFEV1 and ppFVC.<br>• Evaluation of inflammatory markers, including CRP, IgG, IL-8, IL-6, and fecal calprotectin.<br>The original study protocol included additional outcome parameters, but these had to be omitted due to organizational challenges during the COVID-19 lockdown and financial constraints.

Dual use research of concern

Policy information about dual use research of concern

Hazards

Could the accidental, deliberate or reckless misuse of agents or technologies generated in the work, or the application of information presented in the manuscript, pose a threat to:

No	Yes	
☒	☐	Public health
☒	☐	National security
☒	☐	Crops and/or livestock
☒	☐	Ecosystems
☒	☐	Any other significant area

Experiments of concern

Does the work involve any of these experiments of concern:

No	Yes	
☒	☐	Demonstrate how to render a vaccine ineffective
☒	☐	Confer resistance to therapeutically useful antibiotics or antiviral agents
☒	☐	Enhance the virulence of a pathogen or render a nonpathogen virulent
☒	☐	Increase transmissibility of a pathogen
☒	☐	Alter the host range of a pathogen
☒	☐	Enable evasion of diagnostic/detection modalities
☒	☐	Enable the weaponization of a biological agent or toxin
☒	☐	Any other potentially harmful combination of experiments and agents

Plants

Seed stocks

Report on the source of all seed stocks or other plant material used. If applicable, state the seed stock centre and catalogue number. If plant specimens were collected from the field, describe the collection location, date and sampling procedures.

Novel plant genotypes

Describe the methods by which all novel plant genotypes were produced. This includes those generated by transgenic approaches, gene editing, chemical/radiation-based mutagenesis and hybridization. For transgenic lines, describe the transformation method, the number of independent lines analyzed and the generation upon which experiments were performed. For gene-edited lines, describe the editor used, the endogenous sequence targeted for editing, the targeting guide RNA sequence (if applicable) and how the editor was applied.

Authentication

Describe any authentication procedures for each seed stock used or novel genotype generated. Describe any experiments used to assess the effect of a mutation and, where applicable, how potential secondary effects (e.g. second site T-DNA insertions, mosiacism, off-target gene editing) were examined.

ChIP-seq

Data deposition

☐ Confirm that both raw and final processed data have been deposited in a public database such as GEO.

☐ Confirm that you have deposited or provided access to graph files (e.g. BED files) for the called peaks.

Data access links
May remain private before publication.

For "Initial submission" or "Revised version" documents, provide reviewer access links. For your "Final submission" document, provide a link to the deposited data.

Files in database submission

Provide a list of all files available in the database submission.

Genome browser session
(e.g. UCSC)

Provide a link to an anonymized genome browser session for "Initial submission" and "Revised version" documents only, to enable peer review. Write "no longer applicable" for "Final submission" documents.

Methodology

Replicates

Describe the experimental replicates, specifying number, type and replicate agreement.

Sequencing depth

Describe the sequencing depth for each experiment, providing the total number of reads, uniquely mapped reads, length of reads and whether they were paired- or single-end.

Antibodies

Describe the antibodies used for the ChIP-seq experiments; as applicable, provide supplier name, catalog number, clone name, and lot number.

Peak calling parameters

Specify the command line program and parameters used for read mapping and peak calling, including the ChIP, control and index files used.

Data quality

Describe the methods used to ensure data quality in full detail, including how many peaks are at FDR 5% and above 5-fold enrichment.

Software

Describe the software used to collect and analyze the ChIP-seq data. For custom code that has been deposited into a community repository, provide accession details.

Flow Cytometry

Plots

Confirm that:

☐ The axis labels state the marker and fluorochrome used (e.g. CD4-FITC).

☐ The axis scales are clearly visible. Include numbers along axes only for bottom left plot of group (a 'group' is an analysis of identical markers).

☐ All plots are contour plots with outliers or pseudocolor plots.

☐ A numerical value for number of cells or percentage (with statistics) is provided.

Methodology

Sample preparation

Describe the sample preparation, detailing the biological source of the cells and any tissue processing steps used.

Instrument

Identify the instrument used for data collection, specifying make and model number.

Software

Describe the software used to collect and analyze the flow cytometry data. For custom code that has been deposited into a community repository, provide accession details.

Cell population abundance | *Describe the abundance of the relevant cell populations within post-sort fractions, providing details on the purity of the samples and how it was determined.*

Gating strategy | *Describe the gating strategy used for all relevant experiments, specifying the preliminary FSC/SSC gates of the starting cell population, indicating where boundaries between "positive" and "negative" staining cell populations are defined.*

☐ Tick this box to confirm that a figure exemplifying the gating strategy is provided in the Supplementary Information.

Magnetic resonance imaging

Experimental design

Design type | *Indicate task or resting state; event-related or block design.*

Design specifications | *Specify the number of blocks, trials or experimental units per session and/or subject, and specify the length of each trial or block (if trials are blocked) and interval between trials.*

Behavioral performance measures | *State number and/or type of variables recorded (e.g. correct button press, response time) and what statistics were used to establish that the subjects were performing the task as expected (e.g. mean, range, and/or standard deviation across subjects).*

Acquisition

Imaging type(s) | *Specify: functional, structural, diffusion, perfusion.*

Field strength | *Specify in Tesla*

Sequence & imaging parameters | *Specify the pulse sequence type (gradient echo, spin echo, etc.), imaging type (EPI, spiral, etc.), field of view, matrix size, slice thickness, orientation and TE/TR/flip angle.*

Area of acquisition | *State whether a whole brain scan was used OR define the area of acquisition, describing how the region was determined.*

Diffusion MRI ☐ Used ☐ Not used

Preprocessing

Preprocessing software | *Provide detail on software version and revision number and on specific parameters (model/functions, brain extraction, segmentation, smoothing kernel size, etc.).*

Normalization | *If data were normalized/standardized, describe the approach(es): specify linear or non-linear and define image types used for transformation OR indicate that data were not normalized and explain rationale for lack of normalization.*

Normalization template | *Describe the template used for normalization/transformation, specifying subject space or group standardized space (e.g. original Talairach, MNI305, ICBM152) OR indicate that the data were not normalized.*

Noise and artifact removal | *Describe your procedure(s) for artifact and structured noise removal, specifying motion parameters, tissue signals and physiological signals (heart rate, respiration).*

Volume censoring | *Define your software and/or method and criteria for volume censoring, and state the extent of such censoring.*

Statistical modeling & inference

Model type and settings | *Specify type (mass univariate, multivariate, RSA, predictive, etc.) and describe essential details of the model at the first and second levels (e.g. fixed, random or mixed effects; drift or auto-correlation).*

Effect(s) tested | *Define precise effect in terms of the task or stimulus conditions instead of psychological concepts and indicate whether ANOVA or factorial designs were used.*

Specify type of analysis: ☐ Whole brain ☐ ROI-based ☐ Both

Statistic type for inference | *Specify voxel-wise or cluster-wise and report all relevant parameters for cluster-wise methods.*
(See Eklund et al. 2016)

Correction | *Describe the type of correction and how it is obtained for multiple comparisons (e.g. FWE, FDR, permutation or Monte Carlo).*

Models & analysis

n/a	Involved in the study
☐	☐ Functional and/or effective connectivity
☐	☐ Graph analysis
☐	☐ Multivariate modeling or predictive analysis

Functional and/or effective connectivity	*Report the measures of dependence used and the model details (e.g. Pearson correlation, partial correlation, mutual information).*
Graph analysis	*Report the dependent variable and connectivity measure, specifying weighted graph or binarized graph, subject- or group-level, and the global and/or node summaries used (e.g. clustering coefficient, efficiency, etc.).*
Multivariate modeling and predictive analysis	*Specify independent variables, features extraction and dimension reduction, model, training and evaluation metrics.*
